# Dental Pulp Stem Cells: An Attractive Alternative for Cell Therapy in Ischemic Stroke

**DOI:** 10.3389/fneur.2019.00824

**Published:** 2019-08-02

**Authors:** Xiaoyan Lan, Zhengwu Sun, Chengyan Chu, Johannes Boltze, Shen Li

**Affiliations:** ^1^Department of Neurology, Dalian Municipal Central Hospital Affiliated to Dalian Medical University, Dalian, China; ^2^Department of Pharmacy, Dalian Municipal Central Hospital Affiliated to Dalian Medical University, Dalian, China; ^3^School of Life Sciences, University of Warwick, Coventry, United Kingdom

**Keywords:** stem cells, dental pulp stem cells, stroke, brain ischemia, cell therapy

## Abstract

Ischemic stroke is a major cause of disability and mortality worldwide, but effective restorative treatments are very limited at present. Regenerative medicine research revealed that stem cells are promising therapeutic options. Dental pulp stem cells (DPSCs) are autologously applicable cells that origin from the neural crest and exhibit neuro-ectodermal features next to multilineage differentiation potentials. DPSCs are of increasing interest since they are relatively easy to obtain, exhibit a strong proliferation ability, and can be cryopreserved for a long time without losing their multi-directional differentiation capacity. Besides, use of DPSCs can avoid fundamental problems such as immune rejection, ethical controversy, and teratogenicity. Therefore, DPSCs provide a tempting prospect for stroke treatment.

The past decade has witnessed intense advancement and tremendous therapeutic achievements in the ability to diagnose and treat stroke, a cerebrovascular disease of which 87% is ischemic in nature. Nevertheless, stroke remains a major cause of disability, morbidity, and mortality worldwide, and constitutes a major socioeconomic problem (1, 2). Ischemic stroke, due to partially or completely blocked blood flow in a cerebral artery, causes ischemic necrosis of brain tissue seriously impairing the health of affected individuals. The main therapeutic strategy for ischemic stroke is timely recanalization. This can either be achieved by tissue-type plasminogen activator application or mechanical thrombectomy. Particularly the latter can be applied up to 24 h after stroke in patients exhibiting a penumbra, and has revolutionized acute stroke treatment. However, the absolute number of patients qualifying for recanalization remains very low (3, 4). Hence, additional treatment approaches being effective beyond the first hours after stroke onset are urgently required.

Stem cell transplantation is a promising strategy to restore neurological function after stroke (5). Experimental stem cell transplantation in animals showed that numerous cell populations can improve functional recovery by a broad spectrum of mechanisms (6–8). Several kinds of stem cells are currently considered for therapy. These include embryonic stem cells (ESCs), fetal stem/progenitor cells, induced pluripotent stem cells (iPSCs), and adult stem cells. While embryonic or induced pluripotent stem cells exhibit a tremendous differentiation potential, they may also inherit a risk for tumor formation (9). The use of embryonic stem cells or fetal stem/progenitor cells raises ethical concerns. Adult stem cells show a limited proliferation and differentiation potential, but can still be beneficial after stroke due numerous mechanisms beyond tissue restoration. They are further believed to be safer in clinical application and their use is ethically less challenging (9–13).

Recent systematic reviews and meta-analyses on the most prominent adult stem cell therapy candidates, mesenchymal stem cells (MSCs), presented evidence that MSCs improve the outcome after stroke in animals (14) and patients, and confirmed the safety and feasibility of the approach (15). Nevertheless, there is still a lack of adult (stem) cells that can be derived from an autologous source, and may exhibit therapeutic abilities beyond those of MSCs.

## Dental Pulp Stem Cells (DPSCs): A New Source of Adult Stem Cells

**Figure 1:**
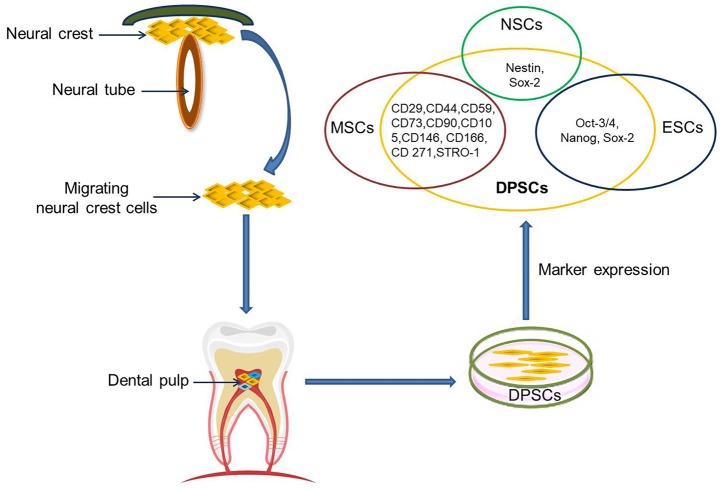
DPSCs origin, isolation, and marker expression. DPSCs originate from migrating neural crest cells, coming to rest in dental pulp, and express markers overlapping with MSCs, ESCs, and NCSs.

The dental pulp is a soft tissue located in the center of teeth. It comprises blood vessels, neural fibers, and connective tissue. The dental pulp contains both mesenchymal and ectodermal tissue as well as neural crest cells (16). Limited dentinal repair in the postnatal organism relies on specialized precursor cell populations residing in the dental pulp tissue. Gronthos et al. first reported the isolation and characterization of stem cells from dental pulp tissue of the third molar in 2000 (17). DPSCs are ectoderm-derived stem cells, originating from migrating neural crest cells (Figure 1). They are a subpopulation among dental pulp cells (DPCs) which possess MSC properties, such as a fibroblast-like morphology, adherence to a plastic surface, as well as surface marker expression, proliferation and colony forming behavior similar to that of MSCs (18, 19). It is not clear whether or not DPSCs are a kind of MSC population. Given their differentiation abilities as reviewed below, it might be assumed that DPSCs are a more naïve stem cell population that also, but not exclusively, exhibits MSC properties. A major benefit of DPSCs is that they can be isolated during routine dental procedures such as the eruption of deciduous teeth or extraction of impacted wisdom teeth (20) in simple and autologous fashion without ethical concerns. Another primary advantage of DPSCs is their potential for cell banking. Several studies have demonstrated that DPSCs retain their stem cell properties after long cryopreservation (21, 22). This is essential as cryopreservation can impact therapeutic capacities of other adult stem cell-containing populations in stroke (23). In addition, DPSC cultures can be established from extracted human molars with high efficiency, even after the whole tooth has been cryopreserved for up to 1 month (24). DPSCs also exhibit a multilineage differentiation potential into chondrocytes, adipocytes, odontoblasts, and potentially even neural-like cells (25–28).

Currently, there are no specific markers that uniquely define DPSCs. In general, DPSCs, as a heterogeneous population, express a variety of markers similar to MSCs (Table 1) (Figure 1), and do not express hematopoietic markers such as CD14, CD19, CD34, and CD45 (18, 26, 29–32). DPSCs isolated by their high proliferative potential tend to include a large population of cells expressing CD44^+^, CD90^+^, and CD166^+^. However, DPSCs also express stemness-related markers similar to ESCs such as Oct-3/4, Nanog, and Sox-2, as well as the cytoskeleton-related markers nestin and vimentin (Figure 1) (33–35). They further express insulin-like growth factor 1 receptor (IGF1R) which is regarded as a pluripotency marker in ESCs. DPSC-secreted IGF1 interacts with IGF1R through an autocrine signaling pathway to maintain self-renewal and proliferation potential (36).

**Table 1:** Characteristics of DPSCs.

	**Marker expression overlapping with**			***In vitro* multipotency**	**Secretomes**
**MSCs**	**ESCs**	**NSCs**	**Mature cells**		
CD29, CD44, CD59, CD73, CD90, CD105, CD146, CD166, CD 271, STRO-1	Oct-3/4, Nanog, Sox-2	Nestin,Sox-2	MAP2, NF, βIII-tubulin, NeuN (neurons), GFAP, S100 (astrocytes), CNPase (oligodendrocytes)	Adipo, chondro, myo, osteo, neuro, odonto	Neuroprotective effect: BDNF, GDNF, VEGF, NGF, IGF-1, PDGF, CNTF, RANTES, GM-CSF; Anti-apoptotic effect: MCP-1, FRACTALKINE; Immunomodulation effect: FLT-3, TGF-β, IL-6

*MAP2, microtubule associated protein 2; NF, Neurofilament; GFAP, glial fibrillary acidic protein; adipo, adipocyte; chondro, chondrocyte; myo, myoblast; neuro, neuronal cell; odonto, odontoblast; osteo, osteoblast. BDNF, brain-derived neurotrophic factor; GDNF, glial cell-derived neurotrophic factor; VEGF, vascular endothelial growth factor; NGF, nerve growth factor; PDGF, platelet derived growth factor; CNTF, ciliary neurotrophic factor, FLT-3, fms-related tyrosine kinase 3; GM-CSF, granulocyte-macrophage colony-stimulating factor; MCP-1, monocyte chemotactic protein 1; TGF-β, transforming growth factor-β; IL-6, interleukin-6*.

In addition, DPSCs (as neural crest-derived stem cells) not only express a number of neural stem cell (NSC) associated markers including nestin (26, 37) and Sox2 (38) (Figure 1), but also express low basal levels of markers associated with mature central nervous system cell types, including the neuronal markers βIII-tubulin, microtubule-associated protein 2 (MAP2), neurofilaments (NF) (33, 39), NeuN (40), the astrocytic marker glial fibrillary acidic protein (GFAP) (26, 33), and oligodendrocyte-associated CNPase (33). Taken together, this suggests that DPSCs can indeed differentiate into neuron-like cells under appropriate conditions, and differentiated cells even exhibit typical electrophysiological properties after neuronal differentiation (41, 42).

## DPSCs as a Potential Candidate for Therapy of Neurological Diseases

Brain-derived NSCs are considered a promising population for stroke treatment due to their ability to self-renew and to differentiate into neural cells types (neurons, astrocytes, oligodendrocytes) (43). However, autologous harvest of adult human NSCs requires neurosurgical procedures due to their brain parenchymal residence (44), while allogeneic or even xenogenic NSCs grafting imposes the risk of graft rejection and additional immunological damage. Only a limited number of clinical trials currently explore the potential of NSCs for stroke treatment because of these limitations.

Adult stem cells or stem cell-containing populations are more frequently applied in translational research. As stated above, DPSCs share many biological characteristics with MSCs including bone marrow MSCs (BM-MSCs), adipose tissue-derived stem cells (ADSCs) and umbilical cord MSCs (UC-MSCs) but there are some variations in their proliferation potential (17, 27, 45), differentiation potential (17, 27, 46), immunomodulatory activity (27), secretome characteristics, and secretory capacity (47–49). Specifically, DPSCs have a higher proliferation rate and a greater clonogenic potential than MSCs (17, 45). Next to DPSCs, the DPC population also contains a higher number of stem/progenitor cells as compared to bone marrow (50). This may be attributed to the developmental state of the respective tissues. All teeth, even the permanent molars, are generated early in individual development and rest in the jar until they erupt. Abilities and capacities of stem cells may be much better preserved in tissue with a slow turnover such as the dental pulp when compared to BM, which exhibits a tremendous turn-over throughout life apart from some niches. DPSCs maintain their high rate of proliferation even after extensive subculturing.

Like MSCs, DPSCs can differentiate into cells of mesenchymal and non-mesenchymal tissues *in vitro* and *in vivo*. However, DPSCs exhibit stronger odontogenesis and neurogenesis capabilities, in turn being not as potent to produce adipogeneic, osteogeneic and chondrogeneic tissue than BM-MSCs (51) and ADSCs (46). Besides, DPSCs also have immunomodulatory capacities exceeding those of BM-MSCs, for example a higher suppression rate of T lymphocyte growth (17, 27).

DPSCs exhibit superior neuroprotective and neuro-supportive properties in neurological injuries and pathologies as compared with BM-MSCs and ADSCs (52). This might be related to a higher expression of trophic factors including brain derived neurotrophic factor (BDNF), glial cell-derived neurotrophic factor (GDNF), nerve growth factor (NGF), vascular endothelial growth factor (VEGF), and platelet derived growth factor (PDGF) in DPSCs as compared to BM-MSCs (47, 48), although the spectrum of growth and trophic factors secretion is similar (53). DPSCs also express higher quantities of CXCL14 and monocyte chemoattractant protein 1 (MCP-1) than ADSCs (49). Besides, the DPSC secretome contains higher concentrations of RANTES, FRACTALKINE, fms-related tyrosine kinase 3 (FLT-3), granulocyte-macrophage colony-stimulating factor (GM-CSF), and MCP-1 than the BM-MSCs secretome (54). DPSCs show higher angiogenic and neurogenic potentials in ectopic transplantation models compared to BM-MSCs and ADSCs, and exhibit the highest migration capacity. Transplantation of DPSCs in a mouse hindlimb ischemia model produced higher blood flow and capillary density than transplantation of BM-MSCs and ADSCs, which being associated with superior recovery of limb movement abilities and reduction of ischemic hindlimb damage (55). DPSCs also mediate stronger anti-apoptotic effects in a microenvironment challenged by oxidative and serum deprivation than BM-MSCs, ADSCs and UC-MSCs (45).

Cell size and diameter are important for safety after intravascular delivery as they are the major, but not the only, determinants of vascular obstruction and complications (56) (Table 2). Previously reported studies showed that the cell diameter of human DPSCs is around 15–16 μm (59), which is comparable to NSCs (57, 58) but slightly smaller than for most MSC populations (57, 60) including human BM-MSCs (61). Still this means that one has to expect a considerable pulmonary passage filtering effect after intravenous delivery, as well as a risk for microembolism after intraarterial administration (62). Hence, thorough investigations identifying the optimal route of DPSC administration by considering safety and efficacy aspects are recommended in DPSC translational research.

**Table 2:** Overview of cell size of cell populations.

**Cell population**	**Cell diameter**	**Cell source**
NSCs (57, 58)	16 μm	Human fetal brain
DPSCs (59)	15–16 μm	Human dental pulp
MSCs (57, 60)	17–18 μm	Bone marrow; adipose tissue; human umbilical cord blood
Hematopoietic stem/progenitor cells (57)	6–10 μm	Bone marrow; peripheral blood; cord blood
Mononuclear cells (57, 60)	7 μm	Bone marrow; peripheral blood; cord blood

## DPSCs for Ischemic Neuronal Damage: *in vitro* Effects

Treatment with immunosorted IGF1R^+^ DPSCs significantly modulates neurite regeneration and anti-inflammation in primary cortical cultures subject to oxygen/glucose deprivation (OGD) (36). DPSCs cultivated on adult mouse hippocampal slices were able to stimulate neurogenesis in both the CA1 zone and at the edges of the hippocampal slices through neurotrophic support *in vitro* (41). Besides, DPSCs can protect primary hippocampal, mesencephalic (63) and dopaminergic neurons (64) from β-amyloid peptide and 6-OHDA induced toxicity, respectively. Furthermore, DPSCs and conditioned medium from DPSCs show superior protective, migratory, and angiogenic effects in OGD-injured astrocytes as compared to BM-MSCs (52, 65). Reducing reactive gliosis, reactive oxygen species production and inflammatory mediators might contribute to this protective effect (52).

## DPSCs Effects After Ischemic Stroke *in vivo*

Human DPSCs can differentiate toward functionally active neurons under appropriate culture conditions (66–68). This comes on top of their bystander effects, indicating that DPSCs might provide enhanced therapeutic capacities in neurological diseases including stroke, Parkinson's disease, Alzheimer's disease, and spinal cord injury (52, 63, 69). To date, there are several preclinical studies demonstrating that DPSCs exert neuroprotective effect resulting in improved functional outcome and reduced infract volumes in rodent stroke models (Table 3) (52, 66, 68, 70–76). No obvious deleterious effects were observed in these studies (66, 68, 71–73, 75), but have not been always explicitly looked for.

**Table 3:** Overview of DPSCs therapy for ischemic stroke animal models.

**Cell type and dose**	**Delivery method**	**Delivery time**	**Animal model**	**Transplantation paradigm**	**Function and mechanisms**	**Primary endpoint and effect sizes**
Human DPSCs; 6 × 10^5^ in 4 μl (68)	Intracerebral (striatum and cortex)	24 h after MCAO	Rat MCAO (2 h)	Xenogeneic	Improved functional recovery; Differentiation into astrocytes; Paracrine effects.	Neuroscore: 35% improve (*p* < 0.05)^*^
Human DPSCs; 4 × 10^6^ in 500 μl (52)	Intravenous (tail vein)	24 h after MCAO	Rat MCAO (2 h)	Xenogeneic	Improved functional recovery and reduced infarct volume; Differentiated into astrocytes and neuron-like cells; Promoted angiogenesis and inhibited astrogliosis.	Infract volume: 44% decrease, (*p* < 0.05); mNSS: 38% improve (*p* < 0.05)^*^
Human DPSCs; 1 × 10^6^ in 1 ml (70)	Intravenous (tail vein)	immediately after MCAO	Rat MCAO (90 min)	Xenogeneic	Reduced the infarct volume and improved the neurological recovery; Inflammation modulation; BBB permeability modulation; Promoted angiogenesis.	Infract volume: 23% decrease (*p* < 0.01); Rotarod test: 108% improve (*p* < 0.01) ^*^; Forelimb grip strength: 54% improve (*p* < 0.05)^*^
Rat DPSCs; 3 × 10^6^ in 300 μl (71)	Intravenous (tail vein)	24 h after MCAO	Rat MCAO (2 h)	Allogeneic Homologous	Enhanced sensorimotor functional recovery; Differentiation into neuronal progenitor cells and neuron-like cells, and triggered neurogenesis.	mNSS: 52% improve (*p* < 0.05)^*^; Adhesive-removal test: 38% improve (*p* < 0.05)^*^
Rat DPSCs; 1 × 10^6^ in 500 μl (72)	Intravenous (tail vein)	24 h after MCAO	Rat MCAO (2 h)	Allogeneic Homologous	Reduced infarct volume and cerebral edema; Differentiated into neuron-like cells	Infract volume: 31% decrease (*p* < 0.05)
Rat DPSCs and dental pulp-derived neurospheres; 1 × 10^6^ in 1 ml (73)	Intravenous (tail vein)	3 h after brain ischemia	Rat severe forebrain ischemia model (11 min)	Allogeneic Homologous	Improved survival rate and cognitive function; Reduced the dead neurons of hippocampus CA1.	Survival rate: 36% improve (*p* < 0.05); Water-maze test: 62% improve (*p* < 0.05)^*^
Human DPSCs; 1 × 10^6^ in 1 ml (74)	Intravenous (tail vein)	immediately and 3 h after MCAO	Rat MCAO (90 min)	Xenogeneic	Reduced ischemic damage and improved functional recovery; Inflammation modulation	Infract volume: 30% decrease (*p* < 0.05); Rotarod test: 97% improve (*p* < 0.01)^*^; Forelimb grip strength: 40% improve (*p* < 0.01)^*^

* *Compare with vehicle-treated stroke animals*.

A number of remarkable improvements were seen in behavioral tests (Table 3), underpinning the considerable effect DPSCs may exert after ischemic stroke. However, many of the behavioral tests used are known for a tendency to overestimate true functional recovery in standard rodent models so future research may also include the use of highly specific behavioral readout systems (77).

During ischemia, neurons are unable to maintain normal transmembrane ion gradient and balance, resulting in cell death by apoptosis, excitatory toxicity, and oxidative stress. Inflammatory reactions contribute to cell death in subacute and even chronic stages what can be exacerbated in the presence of important stroke risk factors (78, 79). These pathophysiological processes are interrelated and can trigger each other, forming a vicious cycle (80, 81). Indeed, neuroinflammation and immune response after stroke have been recognized as key factors contributing to overall brain damage and the extent of neurological deficit (82). The administration of DPSCs during the acute phase of stroke dampens inflammation *in vivo*, and can promote recovery from in post-ischemia/reperfusion brain injury (70). Moreover, intracerebral transplantation of DPSCs or immunosorted IGF1R^+^ DPSCs into the ischemically injured neonatal murine brain significantly increases immunomodulation, enhances poststroke recovery, and promotes neuroplasticity (36, 67). Further, intravenous transplantation of DPSCs or DPSC-derived neurosphere cells significantly ameliorates the impact of global cerebral ischemia, decreases neuronal cell death in the hippocampal CA1 region, improves neuromotor and cognitive function as well as overall survival rates in stroke animals (73). Moreover, intracerebral transplantation of DPSCs also enhanced poststroke functional recovery after brain injury through increasing expression of the anti-apoptotic protein Bcl-2 (67).

Transplanted DPSCs can migrate into the boundary of ischemic areas, and express neural cell and NSC markers such as βIII tubulin, doublecortin (DCX), nestin, and NF (72). The cells' beneficial effects may even be exerted after xenogeneic transplantation as evidenced by a study showing that porcine DPSCs (CD31^−^/CD146^−^ side population cells) injection promotes recovery form motor impairment and reduced infarct volume, promoted migration and differentiation of endogenous NSCs, and finally induced vasculogenesis after stroke in rats (66).

## DPSC Exosomes—Great Opportunities for Cell Therapy Without Cells

The limited survival, differentiation and integration of DPSC-derived cells into the ischemically lesioned brain implies that the functional improvement is more likely mediated through bystander effects rather than cell replacement and differentiation (33). It has been well-documented that the MSC secretome contains a variety of cytokines, chemokines, and growth factors, along with extracellular vesicles (EVs). The most important EVs in MSC-conditioned medium are exosomes, which are of “nano” size (30–100 nm in diameter) (83, 84). EVs play an important role in intercellular communication because they can transfer RNA, micro-RNA, proteins, membrane receptors and even organelles (mitochondria) between cells (85, 86). MSC-derived EVs are of increasing interest since they may have a comparable therapeutic potential to MSCs themselves, but are relatively safer in application, and can pass through the BBB far more easily than cells (87). Studies demonstrated that administration of BM-MSC or ESC-derived exosomes could significantly increases neurogenesis and vasculogenesis, and promotes functional recovery in stroke animal (88, 89).

Likewise, accumulating evidence demonstrated the potent neuroprotective properties of DPSC-derived EVs. An *in vitro* study showed that DPSC-EVs which were grown on laminin-coated microcarriers display neuroprotective properties in 6-OHDA-exposed human dopaminergic neurons (90). DPSC-EVs also reduce cytotoxicity through anti-apoptotic mechanism by upregulating endogenous Bcl-2, and decrease the expression of the pro-apoptotic regulator Bax in Aβ peptide-exposed human neuroblastoma (SH-SY5Y) cells (54). An *in vivo* study showed that exosomes derived from DPSCs have beneficial effects after focal cerebral ischemia in the rat by stimulating angiogenesis and neurogenesis (91). In addition, the therapeutic potential of DPSC-derived conditioned medium (CM) was found to be similar to that of the injection of living cells in animal model of stroke, leading to motor function improvement and infarct volume reduction (76). Moreover, CM from human DPSCs also induced significant neuroprotection, enhanced neuronal sprouting, and reduced neuroinflammation in a mouse model of Alzheimer disease (92). DPSC-derived exosomes were further shown to exert strong anti-inflammatory effects at levels comparable to those of glucocorticoids. They also suppress cathepsin B and matrix metalloproteinase (MMP) activities at the site of inflammation in mice, likely mediated by the transport of annexin A1, phospholipases, and lipid mediators to the site of inflammation (93). Taken as a whole, these studies showed the potential of DPSC-derived exosomes for the treatment of central nervous system disorders.

Investigation of molecules within EVs provides new insight to EV-mediated beneficial mechanism, although determining the exact composition and content of the exosomal content (cargo) produced by different cell types is hard to establish due to inevitable differences regarding the conditions in which the cells are prepared and processed (83). High-throughput mass spectrometry-based analysis of proteins revealed some surface receptors (CD105, CD73, CD29, CD81, and CD44), signaling molecules (many of which are involved in controlling of TGF-β, BMP, MAPK, and PPAR recipient cell signaling pathways), adhesion molecules and MSC-associated markers which may account for the therapeutic potential of MSC-derived EVs (94, 95). Baglio et al. (96) reported a substantial similarity between the most represented miRNAs in ADSC and BM-MSC exosomes, but their relative proportions are different. The top 5 most abundant miRNAs (accounted for 50 % of the total miRNA reads) in ADSC exosomes were miR-486-5p, miR-10a-5p, miR-10b-5p, miR-191-5p, and miR-222-3p, while miR-143-3p, miR-10b-5p, miR-486-5p, miR-22-3p, and miR-21-5p were among the most abundant for BM-MSC exosomes. Besides, exosome libraries were highly enriched in the class of tRNAs, which represented >50 % of total small RNAs in ADSC exosomes and 23–35% in BM-MSC exosomes. However, since the studies of DPSC exosomes are just at the initial stage, there is no exactly content of these exosomes reported.

## Possible Mechanisms of DPSC Therapy for Ischemic Stroke

**Figure 2:**
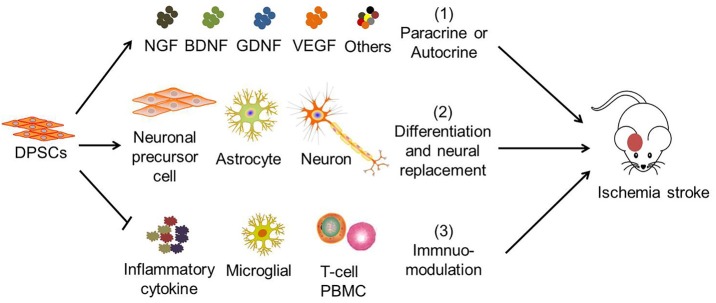
The mechanisms of DPSC therapy for ischemic stroke. The therapeutic effects of DPSCs in stroke are attributed to (1) paracrine or autocrine production of cytokines and growth factors, (2) neural replacement through differentiation into neuronal progenitor cells, astrocytes and neuron-like cells, (3) immuno-modulation with mitigation of pro-inflammatory cytokine expression, of microglial activation, inhibition of activated T-cell response, and peripheral blood mononuclear cell (PBMC) proliferation. (1) and (3) are believed to be the main therapeutic effects of DPSCs.

Previous studies suggested that human DPSCs potentially differentiate into functional neural progenitors or neurons which may integrate into the brain (64, 97, 98). Similarly, studies showed that grafted DPSCs survive, migrated to infarct boundary zones, and differentiate into neurons and astrocytes in the rat. The cells also express neuron-specific markers including βIII tubulin and NF (52, 72). However, only a very small part of transplanted DPSCs (2.3 ± 0.7%) survived in the post-stroke brain, migrated to the peri-infarction areas, and differentiated into astrocytes (51.0 ± 8.6% GFAP^+^) in preference to neurons (8.7 ± 6.1% NeuN^+^) (68). Hence, the therapeutic potential of DPSCs is believed to be mainly exerted by their bystander effects (Figure 2) (76).

DPSCs have the potential to improve the microenvironment and enhancing neurogenesis (47, 64, 99, 100). DPSCs also have been shown to exert potent immune-modulatory properties *via* inhibition of activated T cell responses (27) and peripheral blood mononuclear cell proliferation (101), regulating the expression of inflammatory factors such as TGF-β and interleukin (IL)-6 (102), and the induction of Fas ligand-mediated T cell apoptosis (103). Although the suppression of T cell proliferation seen in *in vitro* studies is now established, this property of DPSCs may not be the sole mechanism of action *in vivo*, particularly since blood circulation increases the number of T cells perpetually. More studies are therefore warranted to further understand the interaction between DPSCs and the immune system because this cross talk has important therapeutic implications.

## Limitations of DPSCs and Challenges Related to Their Use

Though DPSCs have a higher proliferation rate than MSCs, it still needs at least 1 to 2 months to acquire enough cells for therapy from primary isolation (104) (Table 4), which may limit their use at the acute stage of acute onset diseases. The long-term side effects associated with the use of DPSCs also have not been sufficiently studied so far. Further studies are warranted to clarify possible long-term risks associated with the use of these cells, as well as optimal cell preparation, storage and application procedures including routes and time points of application. Tailored potency assays for clinical trials are also lacking, and the optimal route of delivery awaits detailed investigation. Moreover, clinical investigation of the cells has just started (105) so appropriate double-blinded, randomized clinical trials have not yet been reported, currently preventing any conclusion on a potential clinical efficacy of these cells. In addition, further studies thoroughly assessing efficacy, safety and also the content of DPSC-derived EVs are required since EVs are highly promising therapeutic tools for regenerative medicine, but a thorough proof of concept is still missing. Additional research is also needed to capitalize on the DPSC differentiation potential. This might require specialized stroke models, for instance mimicking lacunar stroke, that may be more permissible for tissue restoration, and/or the use of biomaterials to support cell engraftment and survival (106).

**Table 4:** The comparison of DPSCs with other stem cells.

	**DPSCs**	**ESCs**	**NSCs**	**MSCs**		
				**BM-MSCs**	**ADSCs**	**UC-MSCs**
Basic abilities						
Proliferation potential	++	++	+/–	+	+	++
Neurogenic differentiation	+	++	++	+/–	+/–	+/–
Migration abilities	++	++	+	+	+	+
Autologous application abilities	++	−−	–	++	++	–
Required cultivation time to achieve sufficient cell numbers	1–2 months	1–2 months	>2 months	1–2 months	1–2 months	1–2 months
Cryopreservation abilities	+	+	+/–	+	+	+
Cell banking opportunities for adults	+	+/–	–	+/–	+/–	+/–
General amount of information available about cell properties	–	+	+	++	+	+

*(++) very high, (+) high, (+/–) average, (–) low, (−−) very low*.

## Conclusions

This review summarizes the main DPSC characteristics including surface marker expression, proliferation and differentiation potential, cytokine and trophic factor secretion ability, as well as therapeutic effects in *in vitro* and *in vivo* stroke models. It also elucidates important underlying therapeutic mechanisms. DPSCs express a variety of markers that are found on MSCs, ESCs, and NSCs. Although they can differentiate into different types of neuronal cells, bystander effects are believed to be their predominant therapeutic mechanism. DPSCs are widely available, easily accessible, and can support well-established stroke therapies, thereby potentially extending the therapeutic time window and/or augmenting the therapeutic impact. DPSCs differ from the other adult stem cell populations due to their embryonic origin from the neural crest and are of special interest because of their neurotropic character, which makes DPSCs and their exosomes particularly attractive as a new therapeutic tool for the alleviation of symptoms of stroke and, potentially, other neurodegenerative diseases. Besides, DPSCs exhibit a higher proliferation rate, higher expression of trophic factors, stronger neuroprotective effects and neuro-supportive properties *in vitro* and *in vivo* than MSC populations (Table 4), which provide a tempting prospect for stroke treatment.

## Author Contributions

XL, ZS, and CC wrote the manuscript. JB and SL designed the literature assessment strategy and edited the manuscript.

### Conflict of Interest Statement

The authors declare that the research was conducted in the absence of any commercial or financial relationships that could be construed as a potential conflict of interest.
